# Supplementary material for: Multi-scored sleep databases: how to exploit the multiple-labels in automated sleep scoring
Source: Sleep. 2023 Feb 10;46(5):zsad028. doi: 10.1093/sleep/zsad028 (PMC10171642; doi:10.1093/sleep/zsad028)
Supplement: zsad028_suppl_Supplementary_Material [file zsad028_suppl_supplementary_material.docx]

# **SUPPLEMENTARY MATERIAL**

## **Title**

## **Multi-Scored Sleep Databases: How to Exploit the Multiple-Labels in Automated Sleep Scoring**

## Luigi Fiorillo^1,2,*,†^, Davide Pedroncelli^3,†^, Valentina Agostini^3^, Paolo Favaro^1^ and Francesca Dalia Faraci^2^

#### ^1^Institute of Informatics, University of Bern, Bern, Switzerland, ^2^Institute of Digital Technologies for Personalized Healthcare (MeDiTech), Department of Innovative Technologies, University of Applied Sciences and Arts of Southern Switzerland, Lugano, Switzerland, ^3^Department of Electronics and Telecommunications, Politecnico di Torino, Torino, Italy.

##### Institution where work was performed: Institute of Digital Technologies for Personalized Healthcare (MeDiTech), Department of Innovative Technologies, University of Applied Sciences and Arts of Southern Switzerland, Lugano, Switzerland.

##### †These authors contributed equally to this work.

##### *Corresponding author. Luigi Fiorillo, Institute of Digital Technologies for Personalized Healthcare (MeDiTech), Department of Innovative Technologies, University of Applied Sciences and Arts of Southern Switzerland, Lugano, Switzerland. Email: [luigi.fiorillo@supsi.ch](mailto:luigi.fiorillo@supsi.ch).

## **SUPPLEMENTARY ANALYSES**

### **DSN-L**

DSN-L consists of two parallel convolutional neural networks (*CNNs)* branches, with small ${CNN}_{\theta Small}$ and large ${CNN}_{\theta Large}$ filters at the first layer. The convolutional neural networks take as input the 90-second single-channel EEG $\underline{x}_{i}$ signal. The parameters θ of the *CNNs* are independently trained so as to output the two encoding feature vectors $\underline{h}_{i}^{S}$ *(1)* and $\underline{h}_{i}^{L}$ *(2)*. The two vectors are then concatenated in $\underline{f_{i}}$ , *(3)* and forwarded to the final *softmax* layer.

$\underline{h}_{i}^{S}={CNN}_{\theta Small}(\underline{x}_{i} )$ *(1)*

$\underline{h}_{i}^{L}={CNN}_{\theta Large}(\underline{x}_{i} )$ *(2)*

$\underline{f_{i}}=\underline{h}_{i}^{S} || \underline{h}_{i}^{L}$ *(3)*

The *softmax* function (4-5), together with the cross-entropy loss function (6), is used to train the model to output the logits $\underline{z_{i}}$ and the final probabilities of the five mutually exclusive sleep stages classes.

$\underline{z_{i}}=W^{T} \underline{f_{i}}+\underline{b}$ *(4)*

$\hat{p}_{i,k}=\frac{exp (z_{i,k})}{\sum_{j} exp (z_{i,j})}$ *(5)*

$H(\underline{y}_{i} ,\underline{p}_{i}) = \sum_{k=1}^{K} -{y_{i,k}\cdot log( \hat{p}_{i,k})}$ *(6)*

where $\theta=\left\{ W,\underline{b} \right\}$ are the parameters of the *softmax* layer, $j$ is the index of the vector $\underline{z}$, $\hat{p}_{i,k}$ is the output probability of class $k$ of $x(t)$, i.e., the centered 30-second signal in $\underline{x}_{i}$ . In *(6)* we compute the cross-entropy loss to quantify the agreement between the prediction $\underline{p}_{i}$ and the target $\underline{y}_{i}$ (i.e., sleep stage label)**.** For further details we refer the reader to [[1]](#kix.us0ijpl27uhe).

### **SSN**

SSN consists of two main parts: the epoch processing block (*EPB*) and the sequence processing block (*SPB*).

The *EPB* block consists of four modules: (1) spectrogram, (2) signals and frequencies reduction, (3) GRU with attention and (4) positional embedding. In (1) the short-term Fourier transform is computed on each preprocessed epoch, resulting in a time-frequency image $S\in\Re^{C,T,N}$, where $C$ is the number of channels, $T$ is the number of time-steps and $N$ the number of frequency bins. In (2) independent linear projections are applied on the frequencies and channels/signals axis to project $\Re^{C,T,N}$ into $\Re^{c,T,n}$, where $c\leq C$ and $n\leq N$ are the linearly reduced channels and frequencies respectively. In (3) the reshaped $\Re^{T,c,n}$ is the input of the GRU block and the attention layer (implemented as in [[2]](#kix.1c0pktuf8u8w)), and the output is the representation of the sleep epoch in $\Re^{2m_{1}}$, where $m_{1}$ are the hidden units of the GRU layer. In (4) they exploit the positional embedding approach recently proposed in [[3]](#kix.ug5j5jijk1k0) to include the whole night PSG context of each epoch in the following sequence encoder block. First, they build a vector $v=[i_{t}^{epoch}, i_{t,30}^{cycle}, . . . , i_{t,150}^{cycle}]\in\Re^{6}$ for each epoch, where $i_{t}^{epoch}=\frac{t}{1200}$ is the epoch index and $i_{t,l}^{cycle}=cos\left( \frac{t\pi}{l} \right)$ with $l$ in $[30, 60, 90, 120, 150]$ are the cyclic indexes. The vector $v$ is then projected using the Linear+Relu layer to output the positional embedding $i_{t}$ of each epoch. Finally, $i_{t}$ is concatenated with the output of the attention layer to obtain the epoch representation $a_{t}\in\Re^{2m_{1}+6}$.

The *SPB* block consists of two layers of bidirectional gated recurrent unit (GRU) with skip-connections (SkipGRU) and the softmax classification layer. The *SPB* block consists of two layers of bidirectional gated recurrent unit (GRU) with skip-connections (SkipGRU) and the softmax classification layer. The sequence of epochs $a_{1}, . . . ,a_{t}$ is fed to the *SPB* block to output for each epoch the sleep stage probabilities $\hat{p}_{k}\in\Re^{5}$. The softmax function, together with the cross-entropy loss function $H$, is used to train the model to output the probabilities $\hat{p}_{k}$ for the five mutually exclusive classes $K$ that correspond to the five sleep stages. For further details we refer the reader to [[4]](#kix.jtjxtaszj43m).

### **Uncertainty estimate**

We further analyze the ability of the uncertainty estimate and query procedure, proposed in [[1]](#kix.us0ijpl27uhe), to identify the most challenging sleep stage predictions on our calibrated DeepSleepNet-Lite, on both the model trained with and without smoothing their labels, whilst using a $Soft-Consensus$ distribution. We aim at exploring if with a better calibrated model (*i.e.*, the predicted probability value $\hat{p}$ mirrors its ground truth correctness likelihood) we are able to detect a higher number of misclassified epochs.

The query procedure simply relies on the setting of a fixed threshold value $q\%=5\%$, that corresponds to a percentage of sleep epochs to select/reject and to send potentially to the physician for a secondary review. The epochs predicted with the lowest probability values are the $q\%$ selected (on average up to 50 epochs for each PSG recording). In this study we simply use the predicted probability values $\hat{y}_{i}=argmax(\hat{p}_{i,k})$ to select/reject the uncertain sleep epochs.

In [Table S1](#kix.9kv38l6r8i68) we report the overall performance achieved on the DeepSleepNet-Lite models on IS-RC, DOD-H and DOD-O datasets as a result of the above described query procedure. Specifically, the metrics refer to the epochs kept after the $argmax(\hat{p}_{i,k})$ selection procedures ($q\%$ threshold value fixed to $5\%$). We also quantify the percentage of misclassified epochs (%miscl.) among the rejected after the query procedure. The percentage of misclassified epochs is on average in the range 50% to 60%. Consequently, on all the models we have an increase in performance up to 2%-3% in F1-score. These results highlight the efficiency of the query procedure to select a good enough number of misclassified epochs among the selected one. Unlike what we expected, it is not always the case that a better calibrated architecture leads to a better estimate of the model uncertainty. A lower $ECE$ value (see Table 4 in the main manuscript) does not always enable the detection of a significantly higher number of percentages of misclassified epochs (%miscl.).

[1] Fiorillo L, Favaro P, Faraci FD. Deepsleepnet-lite: A simplified automatic sleep stage scoring model with uncertainty estimates. IEEE Transactions on Neural Systems and Rehabilitation Engineering. 2021 Oct 14;29:2076-85.

[2] Luong MT, Pham H, Manning CD. Effective approaches to attention-based neural machine translation. arXiv preprint arXiv:1508.04025. 2015 Aug 17.

[3] Vaswani A, Shazeer N, Parmar N, Uszkoreit J, Jones L, Gomez AN, Kaiser Ł, Polosukhin I. Attention is all you need. Advances in neural information processing systems. 2017;30.

[4] A. Guillot, F. Sauvet, E. H. During, and V. Thorey, “Dreem open datasets: Multi-scored sleep datasets to compare human and automated sleep staging,” IEEE Transactions on Neural Systems and Rehabilitation Engineering, vol. 28, no. 9, pp. 1955–1965, 2020.

## **SUPPLEMENTARY FIGURES**

**Figure S1.** $ACS$ across $\alpha$ values on DSN-L.

$ACS$ values across all the experimented $\alpha$ values, on both the *base+LS_U_* and the *base+LS_SC_* DSN-L based models tested on IS-RC, DOD-H and DOD-O datasets.

**Figure S2.** $ACS$ across $\alpha$ values on SSN.

$ACS$ values across all the experimented $\alpha$ values, on both the *base+LS_U_* and the *base+LS_SC_* SSN based models tested on IS-RC, DOD-H and DOD-O datasets.

## **SUPPLEMENTARY TABLES**

**Table S1.**

Overall performance of the DeepSleepNet-Lite models on IS-RC, DOD-H and DOD-O datasets. The metrics refer to the epochs kept after the $argmax(\hat{p}_{i,k})$ query selection procedures ($q\%$ threshold value fixed to $5\%$). We report the overall accuracy (%Acc.), macro F1-score (%MF1), Cohen’s Kappa (*k*), weighted-averaging F1-score (%F1) and percentage of misclassified epochs among the rejected (%miscl.). The best performance metrics for each dataset are indicated in bold.

|  |  |  | **Overall Metrics** | | | |  |
| --- | --- | --- | --- | --- | --- | --- | --- |
| **Dataset** | **Models** | $\alpha$ | **Acc.** | **MF1** | ***k*** | **F1** | **%miscl.** |
| IS-RC | base | - | 79.2 | 69.6 | 0.69 | 79.7 | 56.7 |
|  | base+LS_U_ | 0.4 | 79.4 | 69.9 | 0.70 | 80.0 | 58.6 |
|  | base+LS_SC_ | 0.6 | **81.6** | **72.0** | **0.72** | **82.0** | **60.2** |
| DOD-H | base | - | 78.6 | 71.6 | 0.70 | 78.8 | 54.4 |
|  | base+LS_U_ | 0.2 | 76.9 | 70.2 | 0.68 | 76.7 | 55.1 |
|  | base+LS_SC_ | 0.8 | **82.1** | **74.3** | **0.74** | **82.3** | **56.1** |
| DOD-O | base | - | 71.2 | 51.7 | 0.58 | 71.6 | **60.4** |
|  | base+LS_U_ | 0.1 | 76.6 | **57.8** | 0.65 | 77.5 | 57.6 |
|  | base+LS_SC_ | 1 | **77.7** | 57.4 | **0.66** | **77.9** | 59.5 |
